# Comparison of Glioblastoma Cell Culture Platforms Based on Transcriptional Similarity with Paired Tissue

**DOI:** 10.3390/ph17040529

**Published:** 2024-04-19

**Authors:** Junseong Park, Ilkyoo Koh, Junghwa Cha, Yoojung Oh, Jin-Kyoung Shim, Hyejin Kim, Ju Hyung Moon, Eui Hyun Kim, Jong Hee Chang, Pilnam Kim, Seok-Gu Kang

**Affiliations:** 1 Department of Neurosurgery, Brain Tumor Center, Severance Hospital, Yonsei University College of Medicine, Seoul 03722, Republic of Korea; j.p@catholic.ac.kr (J.P.); fgmsa98@yuhs.ac (Y.O.); nanjk2@yuhs.ac (J.-K.S.); mjjr80@yuhs.ac (J.H.M.); euihyunkim@yuhs.ac (E.H.K.); changjh@yuhs.ac (J.H.C.); 2 Cancer Evolution Research Center, College of Medicine, The Catholic University of Korea, Seoul 06591, Republic of Korea; 3 Department of Bio and Brain Engineering, KAIST, Daejeon 34141, Republic of Korea; golgyu@kaist.ac.kr (I.K.); cjhwa89@kaist.ac.kr (J.C.); hjkim2800@kaist.ac.kr (H.K.); 4 Brain Tumor Translational Research Laboratory, Severance Biomedical Research Institute, Yonsei University College of Medicine, Seoul 03722, Republic of Korea; 5 KAIST Institute for Health Science and Technology, KAIST, Daejeon 34141, Republic of Korea; 6 Department of Medical Science, Yonsei University Graduate School, Seoul 03722, Republic of Korea

**Keywords:** cell culture platform, extracellular matrix, glioblastoma, patient-derived tumorsphere, transcriptional program

## Abstract

No standardized in vitro cell culture models for glioblastoma (GBM) have yet been established, excluding the traditional two-dimensional culture. GBM tumorspheres (TSs) have been highlighted as a good model platform for testing drug effects and characterizing specific features of GBM, but a detailed evaluation of their suitability and comparative performance is lacking. Here, we isolated GBM TSs and extracellular matrices (ECM) from tissues obtained from newly diagnosed *IDH1* wild-type GBM patients and cultured GBM TSs on five different culture platforms: (1) ordinary TS culture liquid media (LM), (2) collagen-based three-dimensional (3D) matrix, (3) patient typical ECM-based 3D matrix, (4) patient tumor ECM-based 3D matrix, and (5) mouse brain. For evaluation, we obtained transcriptome data from all cultured GBM TSs using microarrays. The LM platform exhibited the most similar transcriptional program to paired tissues based on GBM genes, stemness- and invasiveness-related genes, transcription factor activity, and canonical signaling pathways. GBM TSs can be cultured via an easy-to-handle and cost- and time-efficient LM platform while preserving the transcriptional program of the originating tissues without supplementing the ECM or embedding it into the mouse brain. In addition to applications in basic cancer research, GBM TSs cultured in LM may also serve as patient avatars in drug screening and pre-clinical evaluation of targeted therapy and as standardized and clinically relevant models for precision medicine.

## 1. Introduction

Glioblastoma (GBM), the most common primary brain tumor, is associated with poor prognosis and high mortality [1], despite the application of the best treatment modalities [2]. Although extensive research in this field has resulted in the molecular and prognostic classification of GBM [3,4] and has led to the development of therapeutic agents for GBM, numerous recent clinical trials have failed to significantly improve the prognosis of affected patients, especially those suffering a relapse [5,6]. This gap between pre-clinical and clinical outcomes may stem from the fact that current in vitro studies are frequently conducted in two-dimensional (2D) cell cultures, which do not sufficiently recapitulate the tumor microenvironment (TME) [5,7]. Traditional 2D cell culture platforms using Petri dishes led to clonal selection for fast-growing and culture-compatible cell populations, which entails a loss of cellular diversity and often a homogeneous cell population. Cells cultured in 2D models are unable to grow in all directions due to the flat and stretched morphology enforced by their monolayer arrangement [8]. Furthermore, these cells are adapted to conditions of 20% oxygen, which exceeds the usual oxygen level in tumor tissues of approximately 5% [5,7].

In vitro three-dimensional (3D) cell culture can be used to overcome these problems while maintaining physiological cell–cell and cell–extracellular matrix (ECM) interactions, allowing cells to grow in any direction in a TME that closely resembles in vivo conditions [9,10]. Cells cultured in 3D platforms do not receive homogeneous supplies of oxygen, nutrients, or growth factors due to their large size and the resulting diffusion gradient; this leads to heterogeneous cellular subpopulations, including proliferating, quiescent, and necrotic stages [8,11]. Three-dimensional models are increasingly employed for the in vitro culture of GBM cells. The malignant progression of GBM, characterized by an infiltrative phenotype and resistance to conventional therapies [12], is related to the stem-like cells present at the invasive front [13]. These cells can be isolated from GBM tissues and 3D-cultured in vitro as tumorspheres (TSs) [14]. Accordingly, GBM patient-derived primary TSs are considered good model platforms for testing drug effects and characterizing specific features of GBM, including stemness and invasiveness [15,16].

The interaction between GBM cells and the unique extracellular environment of the brain can affect the diverse characteristics of GBM. Tenascins, fibronectin, fibulin-3, vitronectin, and hyaluronic acid are the primary components of the GBM ECM, which are unregulated at the border of spreading GBM cells [17]. These ECM components can be employed in 3D cell culture to mimic the composition and porosity of the in vivo GBM ECM [8]. For example, decellularized matrices have been used as scaffolds that support ideal cell–matrix interactions [18]. Owing to this distinct composition of the brain ECM compared to other tissues and organs, culture media surrounding 3D-cultured cells are important for preserving the original features of GBM, and further assessment of diverse culture platforms for GBM TSs is therefore necessary.

To address this issue, we compared five different culture platforms for GBM TSs by changing the artificially created or patient-derived microenvironments surrounding GBM TSs: (1) ordinary TS culture liquid media (LM), (2) collagen-based 3D matrix, (3) patient standard ECM (nECM)-based 3D matrix, (4) patient tumor ECM (tECM)-based 3D matrix, and (5) mouse brain. We obtained transcriptome data of GBM TSs cultured via all these culture systems using microarrays and compared them with those obtained from paired GBM tissues. Based on our findings, we propose a culture platform for GBM TSs that preserves the transcriptional program of the original GBM tissues.

## 2. Results

### 2.1. Five Culture Platforms for GBM TSs

**Figure 1:**
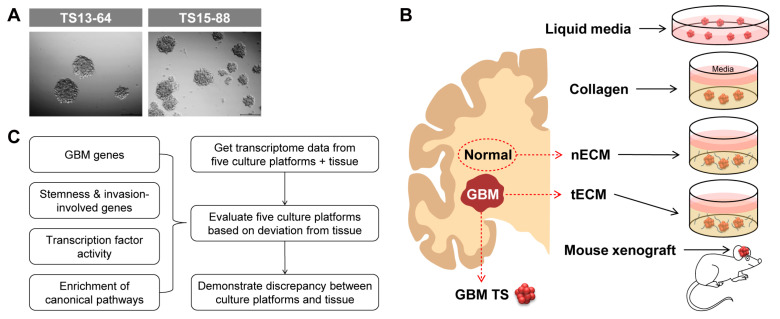
Schematic diagram of the five culture platforms and work flow. (**A**) Representative images of GBM patient-derived TSs. (**B**) Schematic diagram of five culture platforms for GBM TSs: one liquid media culture (LM), three ECM matrix cultures (collagen, nECM, and tECM), and one in vivo culture (mouse xenograft). (**C**) Schematic diagram of the work flow.

We isolated five GBM TSs (TS13-20, TS13-64, TS14-08, TS14-15, and TS15-88) from tumor tissues obtained from patients with newly diagnosed *IDH1* wild-type GBM (Figure 1A) and also isolated nECM and tECM from patients. The entire proteomic composition of patient-derived decellularized ECM was profiled using mass spectrometry. In the overall proteomic profile, numerous components were expressed at higher levels in the tECM than in the nECM. In particular, collagen type 6 family, fibronectin, and tenascin C, which are rarely expressed in normal areas, exhibited prominent expression in the brain tumor tissue (Figure S1). GBM TSs were cultured on five different culture platforms: (1) ordinary TS culture LM, (2) collagen-based 3D matrix, (3) patient nECM-based 3D matrix, (4) patient tECM-based 3D matrix, and (5) mouse brain (Figure 1B). To evaluate each culture platform, we obtained transcriptome data of GBM TSs cultured in all culture systems (one-week culture for (1–4) LM and 3D matrix; immediately after extraction for (5) mouse brain) using microarrays. We evaluated the deviation in the transcriptional program between cultured GBM TSs and paired GBM tissues based on the four aspects, which are GBM genes, stemness- and invasiveness-related genes, transcription factor (TF) activity, and canonical signaling pathways (Figure 1C). Notably, GBM TSs extracted from xenografted mice did not grow in mass, exhibiting core necrosis. Instead, we had to isolate TSs from mouse tumor masses in a manner similar to the protocol using fresh patient GBM tissues, thereby losing characteristics obtained by inoculation into the mouse brain. Since these GBM TSs exhibited very similar phenotypes to the original TSs before xenograft, we did not perform additional experiments using them.

### 2.2. Differential Expression of GBM-Associated Genes among Culture Platforms

**Figure 2:**
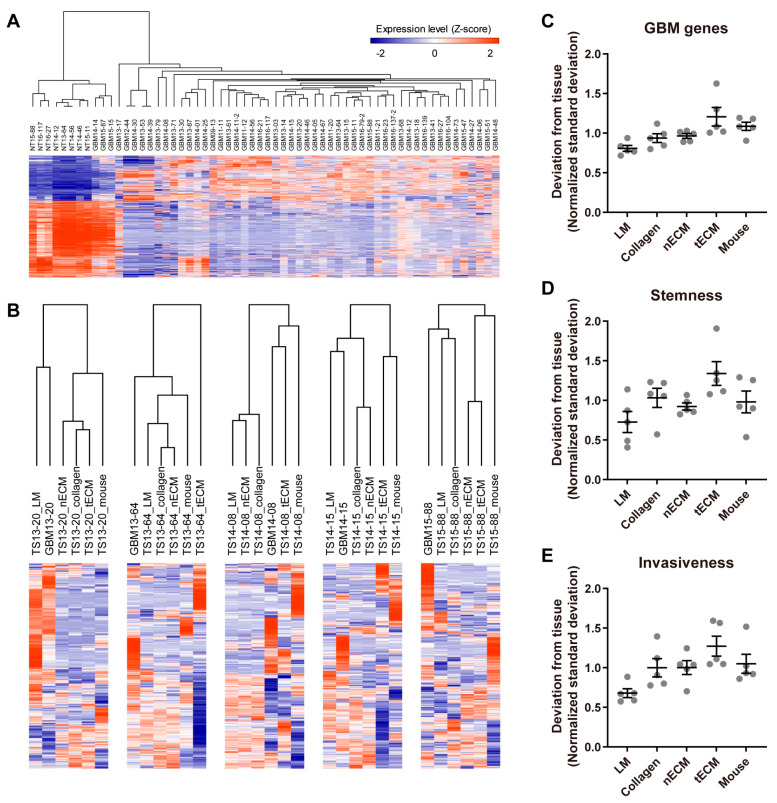
Differential expression of GBM genes by culture platforms. (**A**) Transcriptome data from normal brain tissues (n = 8) and GBM tissues (n = 52) were compared by a two-tailed Student’s *t*-test, and 1244 genes with FDR-corrected q-values < 1 × 10^−15^ were selected as GBM genes. (**B**) For each GBM TS cultured on one of five platforms, unsupervised hierarchical clustering of GBM gene expression was performed using Pearson’s correlation as the distance metric. The dendrogram shows distances among culture platforms. (**C**–**E**) For all GBM genes (**C**), stemness-associated genes (*PROM1*, *NES*, *POU5F1*) (**D**), and invasiveness-associated genes (*ZEB1*, *CTNNB1*, *CDH1*, *CDH2*, *SNAI2*, *TWIST1*, *HAS1*) (**E**), standard deviations of expression levels between each culture platform and tissue were calculated for each sample. For clear visualization, these values were further normalized by samples so that average deviations from tissue for a single sample were 1. Values are displayed as the mean ± SEM. Each dot indicates one of five samples (13-20, 13-64, 14-08, 14-15, 15-88).

To assess the similarity of the transcriptome between each culture platform and GBM tissue, we first identified GBM genes by comparing the transcriptomes of normal and GBM tissues (Figure 2A). Using these 1244 GBM genes (Table S1), unsupervised clustering was performed in each GBM TS, and LM groups tended to cluster closely with the tissue groups (Figure 2B). To quantify the discrepancy between platforms and tissue, standard deviations of the expression levels between each culture platform and tissue were calculated for all GBM genes. Consistent with Figure 2B, the LM group exhibited the lowest normalized standard deviation from the tissue among culture platforms (Figure 2C). Next, we applied the same method using stemness- and invasiveness-associated genes instead of whole GBM genes since these constitute the major phenotypes of GBM TSs. Consistent with the use of whole GBM genes (Figure 2C), GBM TSs cultured on the LM platform exhibited the lowest normalized standard deviation from tissue among culture platforms (Figure 2D,E), suggesting that the LM culture platform maintains a transcriptional program more analogous to GBM tissues than the other four culture platforms.

### 2.3. Differential Activity of TFs among Culture Platforms

**Figure 3:**
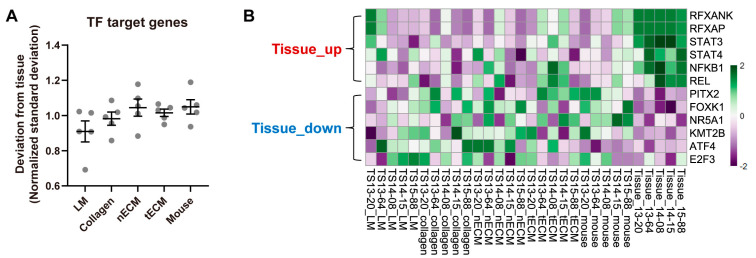
Differential TF activities by culture platforms. (**A**) For all TFs, the standard deviations of GSVA enrichment scores between each culture platform and tissue were calculated for each sample. For clear visualization, these values were further normalized by samples so that the average deviations from tissue for a single sample were 1. Values are displayed as the mean ± SEM. Each dot indicates one of five samples (13-20, 13-64, 14-08, 14-15, 15-88). (**B**) Representative tissue-upregulated and downregulated TFs are presented as a heat map.

To quantify the activity of each TF in the five culture platforms, gene set variation analysis (GSVA) was performed using curated TF target gene sets and the calculated standard deviation of the enrichment scores for all TFs between each culture platform and tissue. Consistent with the results using the expression levels of GBM genes (Figure 2), the LM group exhibited the lowest normalized standard deviation from tissue among culture platforms (Figure 3A), indicating that this platform yielded the most similar overall TF activity with tissues. Nevertheless, GBM tissues still displayed a differential transcriptional program compared to all cultured GBM TSs. Representative upregulated TFs in tissues included STAT3, NFKB1, and REL, whereas downregulated TFs in tissues included ATF4, E2F3, and KMT2B (Figure 3B).

### 2.4. Differential Activity of Canonical Signaling Pathways among Culture Platforms

**Figure 4:**
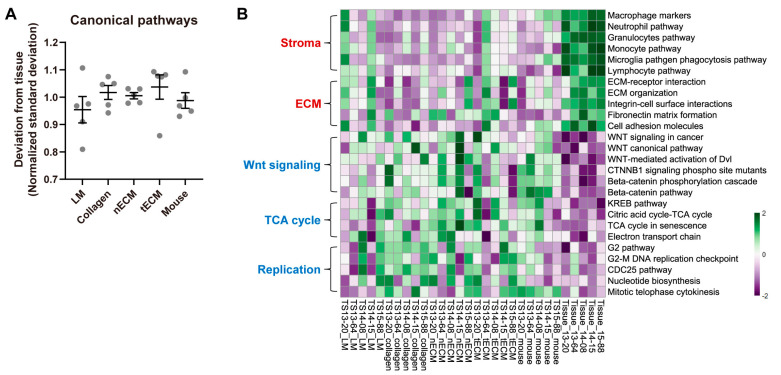
Differential activities of canonical signaling pathways by culture platforms. (**A**) For all canonical signaling pathways, the standard deviations of GSVA enrichment scores between each culture platform and tissue were calculated for each sample. For clear visualization, these values were further normalized by samples so that the average deviations from tissue for a single sample were 1. Values are displayed as the mean ± SEM. Each dot indicates one of five samples (13-20, 13-64, 14-08, 14-15, 15-88). (**B**) Representative tissue-upregulated and downregulated signaling pathways are presented as a heat map.

We also compared each culture platform based on the GSVA enrichment scores for canonical signaling pathways. We calculated the standard deviations of scores between culture platforms and tissues. Consistent with previous analyses (Figure 2 and Figure 3), the LM group exhibited the lowest normalized standard deviation from tissues among culture platforms (Figure 4A), indicating that GBM TSs cultured on this platform showed the most similar signaling pathway activity to tissues. Although the LM culture platform helped GBM TSs preserve the activity of signaling pathways, discrepancies in tissues were still present. The most upregulated signaling pathways in tissues included tumor stroma- or ECM-associated pathways, which are associated with the function of stromal cells rather than components in isolated GBM TSs. In contrast, representative downregulated signaling pathways included Wnt signaling-, TCA cycle-, and cell replication-related pathways, indicating more rapid cell proliferation of GBM TSs than cells in tissues (Figure 4B). However, the LM group showed the most similar enrichment scores to paired tissues among the five culture platforms, even for these signaling pathways.

## 3. Discussion

In this study, we evaluated five different culture platforms for GBM TSs in terms of their transcriptional program. While the brain-specific ECM and its interaction with GBM cells have been extensively investigated [17], no one has yet shown which culture platform is objectively the best. We were able to clearly demonstrate for the first time that the simple LM platform showed the best performance in preserving transcriptional programs, similar to that in paired tissues.

Although it could be assumed that the four other culture platforms are superior to the LM platform, the results differ from such expectations. In addition, although culture platforms using collagen, nECM, or tECM represent a closer equivalent to the 3D tissue microenvironment than the LM platform, in vitro culture is intrinsically distinct from physiological conditions in terms of blood supply, immune infiltration, and crosstalk with diverse signaling molecules [9]. Based on these aspects, several studies previously reported the advantages of ordinary 2D cell culture rather than 3D- or ECM-based culture platforms. Edmonds and Woodruff compared four culture methods for testicular organoid models and showed that 2D ECM and 3D ECM-free media successfully generated organoids, while 3D ECM media failed [19]. Moreover, the organoid-derived 2D monolayer culture method was established in the expanding field of intestinal organoid research to overcome some limitations of 3D organoid culture [20]. This harmonization protocol is increasingly used in infection research to study physiological processes and tissue barrier functions, where easy experimental access of pathogens to the luminal or basolateral cell surface is required [20]. In addition, the pros and cons of diverse in vitro models of liver cell culture, including 2D suspension or monolayer culture with or without ECM components [21,22,23], coculture [24], 3D spheroids [25,26], and decellularized liver biomatrix [27,28,29], were well-summarized [18], suggesting further refinement, optimization, and harmonization of several platforms rather than application of specific ECM-based protocols. All these kinds of literature still indicate the importance of LM-based cell culture models, while many kinds of literature state a paradigm shift from classical 2D monolayer cell cultures to more technically advanced models that allow cell–cell and cell–matrix interactions. Furthermore, GBM TSs already constitute a 3D structure rather than a dish-attached 2D formation without any embedding matrix [14]. Consequently, we speculated that efficient supplementation using LM is more critical than the physical resemblance of matrix-based culture platforms to mimic the transcriptional program of parental tissues. Without supplementing artificially manipulated or patient-derived ECM or embedding them into the mouse brain to imitate the TME of the brain, GBM TSs can be cultured using an easy-to-handle, cost-efficient, and time-saving LM platform while preserving the transcriptional program of the originating tissues.

Although LM well maintained the transcriptional program, some discrepancies between GBM TSs and paired tissues were still detected. Tissue-upregulated TFs, including STAT3, NFKB1, and REL (Figure 3B), are involved in immune responses [30] and GBM invasiveness [31]. Specifically, STAT3 was identified as an invasion-deterministic transcription factor in glioblastoma based on transcriptome analysis of GBM TSs and paired tissues [31]. Furthermore, tissue-upregulated signaling pathways are involved in tumor stroma- and ECM-associated pathways (Figure 4B). All these tissue-enriched gene sets were related to the function of stromal cells rather than cancer cells, and the expression of these genes requires the activation of diverse signaling pathways with crosstalk rather than surrounding ECM conditions [19]. With the exception of LM, which led to the transcriptional program most similar to parental tissues, the surrounding ECM could not activate these pathways. In contrast, the tissue-downregulated signaling pathways included TCA cycle- and cell replication-related pathways. Cell cycle-associated TFs, including E2F3 [32] and KMT2B [33], were consistently downregulated in tissues, implying that GBM TSs proliferate more rapidly than cells in tissues. This can be explained by the clonal selection of proliferating tumor cells during TS isolation from tissues and passaging, as well as the stable in vitro supply of nutrients and oxygen under culture conditions.

Establishing a suitable in vitro cell culture model is important for studying diverse cancer types [34]. Media or the matrix surrounding cells can influence the response of cultured cells to medications by altering their sensitivity to drugs or their mechanism of action [35]. In the absence of clinically available targeted therapies for GBM, the assessment of diverse culture platforms for GBM TSs is especially important. We here used the transcriptional similarity with tissues, which is critical for screening drug efficacy and identifying drug targets, as an evaluation metric [36]. In addition to applications in basic cancer research, GBM TSs cultured in LM may also serve as patient avatars for drug screening and pre-clinical evaluation of targeted therapy. Although they lack the stromal components of TME, such as immune and vascular cells, the LM culture platform for GBM TSs can function as a standardized and clinically relevant model for precision medicine owing to its scalability and reproducibility.

## 4. Materials and Methods

### 4.1. Patient Information and Isolation of GBM TSs

We studied five patients with *IDH1* wild-type GBM who were newly diagnosed with no treatment history via surgery, chemotherapy, or radiotherapy (Table 1). Patient-derived GBM TSs were established from fresh tissue specimens, as previously described [14]. For TS culture in LM [16], cells were cultured in TS complete medium containing DMEM/F-12 (Mediatech, Manassas, VA, USA), 1× B27 (Invitrogen, Waltham, MA, USA), 20 ng/mL bFGF, and 20 ng/mL EGF (Sigma-Aldrich, St. Louis, MO, USA).

### 4.2. Preparation of Patient-Derived ECM

Patient brain tissues were cut into small pieces (3 × 3 × 3 mm) and treated with a decellularizing solution (0.1% (*v*/*v*) ammonium hydroxide (Sigma-Aldrich) and 1% (*v*/*v*) Triton X-100 (Sigma-Aldrich) in distilled water) for 2 days to remove cellular components. Decellularized patient-derived brain ECM (nECM from normal tissue; tECM from tumor tissue) was washed with distilled water to remove the detergent solution and cellular residues. Finally, nECM and tECM were lyophilized and stored at −20 °C until use. For subsequent experiments, lyophilized nECM and tECM were ground and then enzymatically digested with 1 mg/mL pepsin (Sigma-Aldrich) in 0.01N HCl (Sigma-Aldrich) for 2 days at room temperature until visible ECM particles disappeared. The final concentration of the ECM solution was 20 mg/mL. To prepare the hydrogels, the ECM solution was adjusted to neutral pH (7.0) using NaOH (1 M) (Sigma-Aldrich), mixed with 10× PBS, and diluted to the desired final concentration (20 mg/mL) with ice-cold distilled water. The ECM solution was then blended with a collagen solution (4 mg/mL, BD Biosciences, Mississauga, ON, Canada) at a ratio of 10:1 (*v*/*v*). Finally, the pre-gel solution was incubated for 1 h at 37 °C, and GBM TSs were cultured in LM, collagen hydrogel, nECM, and tECM environments. The characterization of nECM and tECM was performed to ensure quality control, as previously described [37].

### 4.3. Protein Extraction from Patient-Derived ECM

Each decellularized patient-derived brain ECM was lysed with 5% sodium dodecyl sulfate (Sigma-Aldrich) in 0.05 M triethylammonium bicarbonate (TEAB; Merck, Rahway, NJ, USA) and quantified using the Pierce™ BCA Protein Assay Kit (Thermo Fisher Scientific, Waltham, MA, USA). Pooled ECM proteins were digested with the S-Trap mini devices (Protifi, Fairport, NY, USA) using the manufacturer’s protocol. Dried peptides were dissolved in 30 μL of 0.1% formic acid.

### 4.4. Nano LC–ESI-MS/MS Analysis

A nano-flow ultra-high-performance liquid chromatography (UHPLC) system (UltiMate 3000; Thermo Fisher Scientific) coupled with an Orbitrap Exploris Tribrid™ mass spectrometer (Thermo Fisher Scientific) was used for all experiments for the analyses of pooled ECM peptides. Samples were injected and separated on EASY-Spray PepMap™ RSLC C18 Column ES803A (2 μm, 100 Å, 75 μm × 50 cm, Thermo Fisher Scientific), operated at 50 °C. A mobile phase B gradient from 5 to 95% was applied over 120 min with a flow rate of 250 nL/min, using H_2_O/FA (99.9:0.1, *v*:*v*) as mobile phase A and acetonitrile/H_2_O/FA (80:19.9:0.1, *v*:*v*:*v*) as mobile phase B. The ESI voltage was 2000 V, and the ion transfer tube temperature was 275 °C.

UHPLC–MS/MS data were acquired based on a data-dependent top-speed mode consisting of a full scan that maximized the number of MS2 scans over 3 s of cycle time. A full scan (MS1) was detected by the Orbitrap analyzer at a resolution of 120 K with a mass range of 400–2000 *m*/*z*. The automatic gain control (AGC) target value was set at standard mode, the maximum injection time was set at automatic mode, and the included charge states were 2–7. The second scan (MS2) was detected by the Orbitrap analyzer at a resolution of 30 K with a fixed collision energy of 30%. The maximum injection time was set to automatic mode, the isolation window size was 1.2 *m*/*z*, the AGC target value was set to standard mode, and the fixed first mass was 110 *m*/*z*.

### 4.5. Proteome Data Analysis

Raw files were converted to MS2 format using RawConverter (The Scripps Research Institute, San Diego, CA, USA). Proteome search was conducted by the Integrated Proteomics Platform (IP2) for MS data analysis (Bruker, Billerica, MA, USA). The following IP2 parameters were used: DBs were whole-reviewed human proteins (UniProt, downloaded in 2022), the precursor/peptide mass tolerance was 20 ppm, the fragment mass tolerance was 20 ppm, and the maximum number of internal missed cleavages was 1. Cysteine residues were searched with a static modification for carboxyaminomethylation, and methionine residues were searched with a variable modification for oxidation. The minimum number of peptides per protein was 2, and the FDR was set to 0.01 at the protein level.

### 4.6. Analysis of Gene Expression Profile

Total RNA was extracted from GBM TSs cultured on each culture platform and the matched patient tissues using a Qiagen RNeasy Plus Mini kit according to the manufacturer’s protocol and loaded onto an Illumina HumanHT-12 v4 Expression BeadChip (Illumina, San Diego, CA, USA). After applying the variance-stabilizing transformation, the data were quantile-normalized using the Bioconductor lumi package in R 4.3.1 [38]. Using GENE-E software (http://software.broadinstitute.org/GENE-E/ (accessed on 17 April 2024)), hierarchical clustering was performed with Pearson’s correlation as a distance metric (average linkage), and expression levels were depicted as heat maps. Gene set enrichment analysis was performed using the R package GSVA (1.50.2) [39] with gene sets for TF target genes [40] and MSigDB C2 canonical pathways.

### 4.7. Mouse Orthotopic Xenograft Model

Male athymic nude mice (6 weeks old; Central Lab. Animal Inc., Seoul, Republic of Korea) were housed in micro-isolator cages under sterile conditions and observed for at least one week before study initiation to ensure proper health. The mice were collectively housed in laboratory cages, with each cage accommodating five individuals. The mice had free access to food and water and were kept in a room with a controlled temperature (22 ± 2 °C) and humidity (55 ± 5%) under a 12 h light/dark cycle. The operational procedures were carried out under deep anesthesia and analgesia via intraperitoneal injection of a solution containing Zoletil (30 mg/kg) and xylazine (10 mg/kg) [41]. No side effects were observed in any mice after the administration of anesthetics. Dissociated GBM TSs in TS complete medium (5 × 10^5^ cells per mouse; n = 5 mice per patient-derived TSs) were injected into the right frontal lobe at a depth of 4.5 mm using a guide-screw system [42]. If the body weight decreased by more than 15% relative to the maximum weight, mice were euthanized according to the 2020 guidelines of the American Veterinary Medical Association. Euthanasia was achieved by charging the CO_2_ chamber at a flow rate of 50–70% per minute and proceeding within the cages to minimize stress for the mice. After euthanasia, the cessation of cardiac activity was verified to confirm the complete cessation of life. The formation of tumor mass was confirmed via H&E staining immediately after euthanasia of each mouse, and extracted tumor tissues were subjected to microarray experiments to obtain transcriptome data.

## 5. Conclusions

Establishing a suitable in vitro cell culture model is important for studying diverse cancer types. In this study, we evaluated five different culture platforms for GBM TSs by changing the artificially created or patient-derived microenvironments surrounding them, as follows: (1) TS culture LM, (2) collagen-based 3D matrix, (3) patient nECM-based 3D matrix, (4) patient tECM-based 3D matrix, and (5) mouse brain. Our results clearly indicate that the simple LM platform showed the best performance in terms of preserving transcriptional programs, similar to that of paired tissues. Owing to this advantage, as well as their scalability and reproducibility, GBM TSs cultured in LM can function as a standardized and clinically relevant model for basic cancer research and precision medicine. Furthermore, they may serve as patient avatars for drug screening and pre-clinical evaluation of targeted therapy.

## Figures and Tables

**Table 1:** Clinical characteristics of TS-matched GBM patients.

Case	Sex	Age	*IDH1* Mutation	*MGMT* Promoter	1p/19q Deletion
13-20	M	61	Wild-type	Methylated	Intact
13-64	F	56	Wild-type	Unmethylated	Intact
14-08	F	57	Wild-type	Unmethylated	Intact
14-15	M	67	Wild-type	Methylated	Intact
15-88	M	61	Wild-type	Unmethylated	Intact

## Data Availability

Microarray datasets are available in the Gene Expression Omnibus repository: GSE249289, GSE159000, and GSE131837 [3]. Mass spectrometry datasets are available in ProteomeXchange (PXD047560) and MassIVE (MSV000093581).

## Supplementary Materials

The following supporting information can be downloaded at https://www.mdpi.com/article/10.3390/ph17040529/s1, Figure S1: Differentially expressed proteins between nECM and tECM; Table S1: The list of GBM genes.

## References

[1] Hoshide R., Jandial R. (2016). 2016 World Health Organization Classification of Central Nervous System Tumors: An Era of Molecular Biology. World Neurosurg..

[2] Stupp R., Hegi M.E., Mason W.P., van den Bent M.J., Taphoorn M.J., Janzer R.C., Ludwin S.K., Allgeier A., Fisher B., Belanger K. (2009). Effects of radiotherapy with concomitant and adjuvant temozolomide versus radiotherapy alone on survival in glioblastoma in a randomised phase III study: 5-year analysis of the EORTC-NCIC trial. Lancet Oncol..

[3] Park J., Shim J.K., Yoon S.J., Kim S.H., Chang J.H., Kang S.G. (2019). Transcriptome profiling-based identification of prognostic subtypes and multi-omics signatures of glioblastoma. Sci. Rep..

[4] Wang Q., Hu B., Hu X., Kim H., Squatrito M., Scarpace L., deCarvalho A.C., Lyu S., Li P., Li Y. (2017). Tumor Evolution of Glioma-Intrinsic Gene Expression Subtypes Associates with Immunological Changes in the Microenvironment. Cancer Cell.

[5] Riedel N.C., de Faria F.W., Alfert A., Bruder J.M., Kerl K. (2022). Three-Dimensional Cell Culture Systems in Pediatric and Adult Brain Tumor Precision Medicine. Cancers.

[6] Bagley S.J., Kothari S., Rahman R., Lee E.Q., Dunn G.P., Galanis E., Chang S.M., Nabors L.B., Ahluwalia M.S., Stupp R. (2022). Glioblastoma Clinical Trials: Current Landscape and Opportunities for Improvement. Clin. Cancer Res..

[7] LeSavage B.L., Suhar R.A., Broguiere N., Lutolf M.P., Heilshorn S.C. (2022). Next-generation cancer organoids. Nat. Mater..

[8] Wanigasekara J., Cullen P.J., Bourke P., Tiwari B., Curtin J.F. (2023). Advances in 3D culture systems for therapeutic discovery and development in brain cancer. Drug Discov. Today.

[9] Jensen C., Teng Y. (2020). Is It Time to Start Transitioning From 2D to 3D Cell Culture?. Front. Mol. Biosci..

[10] Pampaloni F., Reynaud E.G., Stelzer E.H. (2007). The third dimension bridges the gap between cell culture and live tissue. Nat. Rev. Mol. Cell Biol..

[11] Alzeeb G., Metges J.P., Corcos L., Le Jossic-Corcos C. (2020). Three-Dimensional Culture Systems in Gastric Cancer Research. Cancers.

[12] Auffinger B., Spencer D., Pytel P., Ahmed A.U., Lesniak M.S. (2015). The role of glioma stem cells in chemotherapy resistance and glioblastoma multiforme recurrence. Expert Rev. Neurother..

[13] Jackson M., Hassiotou F., Nowak A. (2015). Glioblastoma stem-like cells: At the root of tumor recurrence and a therapeutic target. Carcinogenesis.

[14] Kang S.G., Cheong J.H., Huh Y.M., Kim E.H., Kim S.H., Chang J.H. (2015). Potential use of glioblastoma tumorsphere: Clinical credentialing. Arch. Pharm. Res..

[15] Patrizii M., Bartucci M., Pine S.R., Sabaawy H.E. (2018). Utility of Glioblastoma Patient-Derived Orthotopic Xenografts in Drug Discovery and Personalized Therapy. Front. Oncol..

[16] Park J., Shim J.K., Kang J.H., Choi J., Chang J.H., Kim S.Y., Kang S.G. (2018). Regulation of bioenergetics through dual inhibition of aldehyde dehydrogenase and mitochondrial complex I suppresses glioblastoma tumorspheres. Neuro. Oncol..

[17] Mohiuddin E., Wakimoto H. (2021). Extracellular matrix in glioblastoma: Opportunities for emerging therapeutic approaches. Am. J. Cancer Res..

[18] Damania A., Jain E., Kumar A. (2014). Advancements in in vitro hepatic models: Application for drug screening and therapeutics. Hepatol. Int..

[19] Edmonds M.E., Woodruff T.K. (2020). Testicular organoid formation is a property of immature somatic cells, which self-assemble and exhibit long-term hormone-responsive endocrine function. Biofabrication.

[20] Warschkau D., Delgado-Betancourt E., Holthaus D., Muller A., Kliem G., Krug S.M., Schulzke J.D., Aebischer T., Klotz C., Seeber F. (2022). From 3D to 2D: Harmonization of Protocols for Two-dimensional Cultures on Cell Culture Inserts of Intestinal Organoids from Various Species. Bio. Protoc..

[21] Soldatow V.Y., Lecluyse E.L., Griffith L.G., Rusyn I. (2013). In vitro models for liver toxicity testing. Toxicol. Res..

[22] Bissell D.M., Caron J.M., Babiss L.E., Friedman J.M. (1990). Transcriptional regulation of the albumin gene in cultured rat hepatocytes. Role of basement-membrane matrix. Mol. Biol. Med..

[23] Bissell D.M., Arenson D.M., Maher J.J., Roll F.J. (1987). Support of cultured hepatocytes by a laminin-rich gel. Evidence for a functionally significant subendothelial matrix in normal rat liver. J. Clin. Investig..

[24] Bhatia S.N., Balis U.J., Yarmush M.L., Toner M. (1999). Effect of cell-cell interactions in preservation of cellular phenotype: Cocultivation of hepatocytes and nonparenchymal cells. FASEB J..

[25] Du Y., Chia S.M., Han R., Chang S., Tang H., Yu H. (2006). 3D hepatocyte monolayer on hybrid RGD/galactose substratum. Biomaterials.

[26] Nyberg S.L., Hardin J., Amiot B., Argikar U.A., Remmel R.P., Rinaldo P. (2005). Rapid, large-scale formation of porcine hepatocyte spheroids in a novel spheroid reservoir bioartificial liver. Liver Transpl..

[27] Barakat O., Abbasi S., Rodriguez G., Rios J., Wood R.P., Ozaki C., Holley L.S., Gauthier P.K. (2012). Use of decellularized porcine liver for engineering humanized liver organ. J. Surg. Res..

[28] Zhou P., Lessa N., Estrada D.C., Severson E.B., Lingala S., Zern M.A., Nolta J.A., Wu J. (2011). Decellularized liver matrix as a carrier for the transplantation of human fetal and primary hepatocytes in mice. Liver Transpl..

[29] Baptista P.M., Siddiqui M.M., Lozier G., Rodriguez S.R., Atala A., Soker S. (2011). The use of whole organ decellularization for the generation of a vascularized liver organoid. Hepatology.

[30] Danova K., Klapetkova A., Kayserova J., Sediva A., Spisek R., Jelinkova L.P. (2015). NF-kappaB, p38 MAPK, ERK1/2, mTOR, STAT3 and increased glycolysis regulate stability of paricalcitol/dexamethasone-generated tolerogenic dendritic cells in the inflammatory environment. Oncotarget.

[31] Park J., Shim J.K., Lee M., Kim D., Yoon S.J., Moon J.H., Kim E.H., Park J.Y., Chang J.H., Kang S.G. (2023). Classification of IDH wild-type glioblastoma tumorspheres into low- and high-invasion groups based on their transcriptional program. Br. J. Cancer.

[32] Leone G., DeGregori J., Yan Z., Jakoi L., Ishida S., Williams R.S., Nevins J.R. (1998). E2F3 activity is regulated during the cell cycle and is required for the induction of S phase. Genes Dev..

[33] Pang L., Tian H., Gao X., Wang W., Wang X., Zhang Z. (2021). KMT2D deficiency disturbs the proliferation and cell cycle activity of dental epithelial cell line (LS8) partially via Wnt signaling. Biosci. Rep..

[34] Weiswald L.B., Bellet D., Dangles-Marie V. (2015). Spherical cancer models in tumor biology. Neoplasia.

[35] Poornima K., Francis A.P., Hoda M., Eladl M.A., Subramanian S., Veeraraghavan V.P., El-Sherbiny M., Asseri S.M., Hussamuldin A.B.A., Surapaneni K.M. (2022). Implications of Three-Dimensional Cell Culture in Cancer Therapeutic Research. Front. Oncol..

[36] Park J., Lee J., Choi C. (2015). Evaluation of drug-targetable genes by defining modes of abnormality in gene expression. Sci. Rep..

[37] Koh I., Cha J., Park J., Choi J., Kang S.G., Kim P. (2018). The mode and dynamics of glioblastoma cell invasion into a decellularized tissue-derived extracellular matrix-based three-dimensional tumor model. Sci. Rep..

[38] Du P., Kibbe W.A., Lin S.M. (2008). lumi: A pipeline for processing Illumina microarray. Bioinformatics.

[39] Hanzelmann S., Castelo R., Guinney J. (2013). GSVA: Gene set variation analysis for microarray and RNA-seq data. BMC Bioinform..

[40] Garcia-Alonso L., Holland C.H., Ibrahim M.M., Turei D., Saez-Rodriguez J. (2019). Benchmark and integration of resources for the estimation of human transcription factor activities. Genome Res..

[41] Khokhlova O.N., Borozdina N.A., Sadovnikova E.S., Pakhomova I.A., Rudenko P.A., Korolkova Y.V., Kozlov S.A., Dyachenko I.A. (2022). Comparative Study of the Aftereffect of CO(2) Inhalation or Tiletamine-Zolazepam-Xylazine Anesthesia on Laboratory Outbred Rats and Mice. Biomedicines.

[42] Lal S., Lacroix M., Tofilon P., Fuller G.N., Sawaya R., Lang F.F. (2000). An implantable guide-screw system for brain tumor studies in small animals. J. Neurosurg..

