# Supplementary material for: Comparison of Glioblastoma Cell Culture Platforms Based on Transcriptional Similarity with Paired Tissue
Source: Pharmaceuticals (Basel). 2024 Apr 19;17(4):529. doi: 10.3390/ph17040529 (PMC11054899; doi:10.3390/ph17040529)
Supplement: Supplementary file 1 [file pharmaceuticals-17-00529-s001.zip › Supplementary Figure S1.pdf]

Supplementary Material

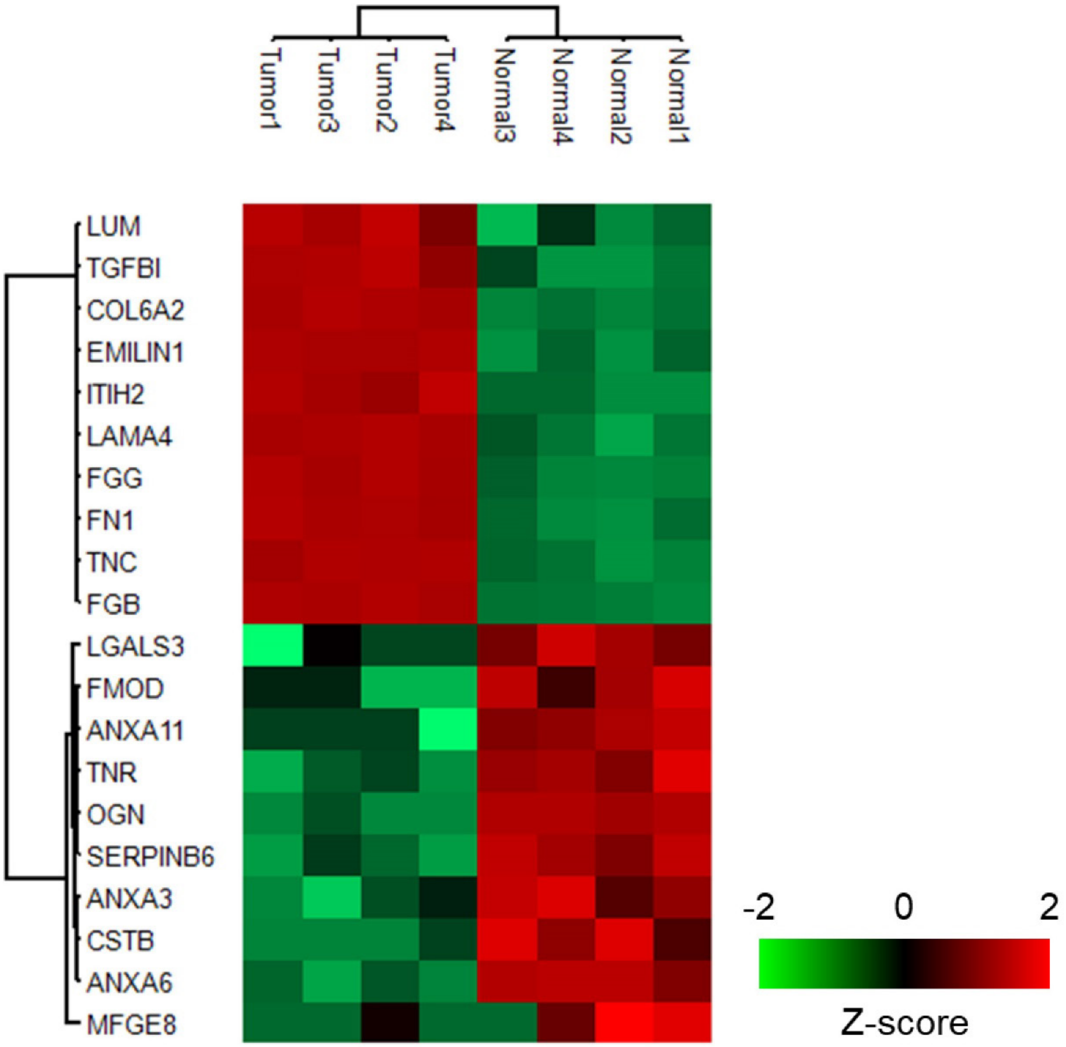

Figure S1. Differentially expressed proteins between nECM and tECM. Quantification of entire proteomic composition of patient-derived decellularized ECM through mass spectrometry.
